# Comparison of sugar content for ionic liquid pretreated Douglas-fir woodchips and forestry residues

**DOI:** 10.1186/1754-6834-6-61

**Published:** 2013-05-01

**Authors:** Aaron M Socha, Samuel P Plummer, Vitalie Stavila, Blake A Simmons, Seema Singh

**Affiliations:** 1 Deconstruction Division, Joint BioEnergy Institute, 5885 Hollis Avenue, Emeryville, CA 94608, USA; 2 Biological and Materials Science Center, Sandia National Laboratories, 7011 East Avenue, Livermore, CA 94551, USA; 3 Department of Chemistry and Chemical Technology, Bronx Community College, Bronx, NY 10453, USA

**Keywords:** Lignocellulose, Biomass pretreatment, Ionic liquid pretreatment, Douglas-fir, Softwood, Woodchips, Forestry residues, 1-ethyl-3-methylimidazolium acetate

## Abstract

**Background:**

The development of affordable woody biomass feedstocks represents a significant opportunity in the development of cellulosic biofuels. Primary woodchips produced by forest mills are considered an ideal feedstock, but the prices they command on the market are currently too expensive for biorefineries. In comparison, forestry residues represent a potential low-cost input but are considered a more challenging feedstock for sugar production due to complexities in composition and potential contamination arising from soil that may be present. We compare the sugar yields, changes in composition in Douglas-fir woodchips and forestry residues after pretreatment using ionic liquids and enzymatic saccharification in order to determine if this approach can efficiently liberate fermentable sugars.

**Results:**

These samples were either mechanically milled through a 2 mm mesh or pretreated as received with the ionic liquid (IL) 1-ethyl-3-methylimidazolium acetate [C_2_mim][OAc] at 120°C and 160°C. IL pretreatment of Douglas-fir woodchips and forestry residues resulted in approximately 71-92% glucose yields after enzymatic saccharification. X-ray diffraction (XRD) showed that the pretreated cellulose was less crystalline after IL pretreatment as compared to untreated control samples. Two-dimensional nuclear magnetic resonance spectroscopy (2D-NMR) revealed changes in lignin and hemicellulose structure and composition as a function of pretreatment. Mass balances of sugar and lignin streams for both the Douglas-fir woodchips and forestry residues throughout the pretreatment and enzymatic saccharification processes are presented.

**Conclusions:**

While the highest sugar yields were observed with the Douglas-fir woodchips, reasonably high sugar yields were obtained from forestry residues after ionic liquid pretreatment. Structural changes to lignin, cellulose and hemicellulose in the woodchips and forestry residues of Douglas-fir after [C_2_mim][OAc] pretreatment are analyzed by XRD and 2D-NMR, and indicate that significant changes occurred. Irrespective of the particle sizes used in this study, ionic liquid pretreatment successfully allowed high glucose yields after enzymatic saccharification. These results indicate that forestry residues may be a more viable feedstock than previously thought for the production of biofuels.

## Results and discussion

### Introduction

Forestry residues comprise parts of trees unsuitable for sawmills. This heterogeneous feedstock includes branches, treetops, small-diameter wood, dead wood, stumps, undeveloped trees and low-value species. In 2012, it is estimated that 50 million dry tons of primary forestry residues are available for less than $40/ton in the United States, a volume projected to remain constant through 2030 [1]. If converted to biofuel this feedstock could displace approximately 1.5% of the nation’s petroleum-based transportation fuels [2]. Pretreatment with the ionic liquid (IL), 1-ethyl-3-methylimidazolium acetate [C_2_mim][OAc] is recognized as a non-toxic, biodegradable [3], highly effective method for decrystallizing cellulose, liberating it from lignin and hemicellulose [4]. When pretreated with [C_2_mim][OAc] a suite of feedstocks, such as softwoods [5], hardwoods [6,7], grasses [8-10], and agricultural wastes [11,12] are converted to biomass that is amenable to enzymatic saccharification and downstream fermentation [13,14].

Due to its predominance in the Pacific Northwest [15,16] we examined the affects of [C_2_mim][OAc] pretreatment of Douglas-fir (*Pseudotsuga menziesii*). As compared to grasses, softwoods are richer in glucan, and as compared to other softwoods such as pine, Douglas-fir has a large percentage of its hemicellulose as mannan [17-19]. Previous studies using dilute acid [19] and sulfur dioxide [18] pretreatment for Douglas-fir have resulted in high glucose recoveries due to a large percentage of hemicellulose solubilization. However both of these methods require high severity for optimal sugar yields and result in the production of compounds inhibitory to downstream fermentation [20,21]. The following study represents the first analysis of IL pretreatment on both Douglas-fir and softwood forestry residues.

The goal of this study was to compare the effects of IL pretreatment, using [C_2_mim][OAc], on woodchips and forestry residues of Douglas-fir in order to determine if the lower cost residues are a viable feedstock in terms of sugar yield and conversion efficiency. Nguyen et al. showed that dilute sulfuric acid pretreatment of 2 mm-milled Douglas-fir solubilizes approximately 91% of the hemicellulose fraction, and allows for approximately 85% of cellulose to be enzymatically converted to glucose [19]. Unfortunately, the conditions required to achieve these yields resulted in the production of furfural and HMF in concentrations inhibitory to downstream fermentation, and thus the method may require a two-stage pretreatment, adding additional costs and time to the process. Interestingly, Boussaid et al. showed that mechanical refining of SO_2_ steam exploded Douglas-fir woodchips decreased its glucose yield from enzymatic hydrolysis, a report contradictory to previous findings in their group [22].

**Figure 1:**
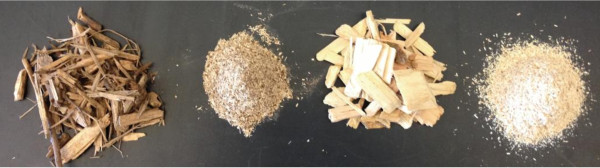
Biomass samples used in this study (from left to right) forestry residues (un-milled), forestry residues (2 mm-milled), Douglas-fir woodchips (un-milled) and Douglas-fir woodchips (2 mm-milled).

To investigate the effects of mechanical refining and pretreatment severity on both Douglas-fir woodchips and forestry residues, we used a 4 × 2 approach. The woodchip and residue feedstocks were Willey-milled to a 2 mm powder or pretreated “un-milled” (Figure 1). Additionally, two temperature severities, 120°C and 160°C, were compared in terms of compositional analysis and enzymatic saccharification. Our results are corroborated by spectroscopic analyses including X-Ray Diffraction (XRD) and 2-Dimensional Nuclear Magnetic Resonance (NMR) spectroscopy.

### Compositional analysis of untreated and IL pretreated Douglas-fir samples

Compositional analysis of the cellulose, hemicellulose and lignin was performed directly on the untreated biomass. Analysis of IL pretreated samples was performed on biomass precipitated from reactions using water as the antisolvent. Sugar and lignin values from untreated samples (i.e. fresh biomass) of Douglas-fir Woodchips were in good agreement with previously published studies [17,18]. Untreated Woodchips were observed to contain 40.0% glucan, 14.3% mannan, 2.2% xylan, 3.2% galactan, 2.2% arabinan, 24.6% lignin and < 1% ash. Approximately 14% of the biomass could not be accounted for, and this most likely results from a combination of sampling error and/or the presence of extractive components such as organic acids, terpenes, phenolics or sugars that were not detected using our analytical methods. As expected, increasing pretreatment temperature resulted in lower solids recovery. We hypothesize that due to greater bulk surface area the 2 mm-milled woodchips and forestry residues were more easily solubilized by [C_2_mim][OAc] than the courser samples. This is evident by comparison of solids recovery for the 2 mm milled vs. un-milled samples in Table 1. The average recovery of all 2 mm-milled samples was 65.8% while that of the un-milled samples was 73.0%.

**Table 1:** Compositional analysis of Douglas-fir Woodchips and forestry residues

**Feedstock**	**Treatment (particle size)**	**Solid recovery, %**	**Glucan, %**	**Mannan, %**	**Xylan, %**	**Galactan, %**	**Arabinan, %**	**Lignin, %**
**Woodchips**	Untreated	------	40.0 ± 0.5	14.3 ± 1.8	2.2 ± 0.1	3.2 ± 0.5	2.2 ± 0.6	24.6 ± 1.2
	120 (2 mm)	70 ± 2.3	48.8 ± 2.7	12.5 ± 2.3	1.7 ± 1.0	2.6 ± 0.3	0.7 ± 0.1	21.6 ± 2.8
	120 (un-milled)	80 ± 2.9	45.9 ± 3.4	13.6 ± 0.4	1.7 ± 0.6	3.1 ± 0.6	0.6 ± 0.4	26.6 ± 3.4
	160 (2 mm)	67 ± 3.6	52.8 ± 2.9	6.0 ± 2.7	1.0 ± 0.2	2.8 ± 0.3	0.5 ± 0.1	19.7 ± 1.3
	160 (un-milled)	70 ± 2.3	50.8 ± 1.9	8.0 ± 1.5	1.2 ± 0.2	2.6 ± 0.3	0.3 ± 0.2	28.5 ± 0.5
**Forestry residues**	Untreated	-	35.4 ± 5.9	10.1 ± 2.2	2.5 ± 0.9	3.5 ± 0.4	0.5 ± 0.1	26.8 ± 1.3
	120 (2 mm)	68 ± 2.8	37.0 ± 3.4	6.3 ± 0.4	2.9 ± 1.1	3.9 ± 0.2	0.6 ± 0.2	30.7 ± 3.1
	120 (un-milled)	77 ± 4.0	36.5 ± 1.7	5.1 ± 0.4	1.9 ± 1.2	3.7 ± 0.7	0.4 ± 0.2	28.4 ± 1.0
	160 (2 mm)	58 ± 2.7	38.3 ± 3.5	4.4 ± 0.8	1.5 ± 0.4	2.9 ± 0.2	0.1 ± 0.2	32.8 ± 1.0
	160 (un-milled)	65 ± 3.4	44.9 ± 2.9	3.7 ± 0.6	1.6 ± 0.2	3.0 ± 0.3	0.1 ± 0.2	30.6 ± 0.1

Increasing pretreatment severity from 120°C to 160°C increased the percent by mass of cellulose through solubilization of hemicellulose and lignin. When compared to IL pretreatment of other softwoods, these results were not surprising. Torr et al. showed a 4.9% increase of glucan alongside a 4.1% decrease in mannan in pine pretreated with [C_2_mim][OAc] for 3 h at 155°C as compared to the same biomass pretreated at 120°C [5]. In experiments with Douglas-fir woodchips, the average glucan increase from 120°C to 160°C was 4.4%, accompanied by a mannan decrease of 6.0%. Upon pretreatment at 120°C, 2 mm-milled and un-milled Douglas-fir woodchips showed an 85.4% and 91.8% glucan recovery, respectively, while also showing reduction in hemicellulose sugars such as mannan (38.8% and 23.9%, respectively) and xylan (45.9% and 38.1%, respectively). When severity was increased to 160°C the 2 mm-milled and the un-milled Douglas-fir woodchip samples showed 88.4% and 88.9% glucan recovery, respectively, with a substantial concomitant decrease in hemicellulose sugars such as mannan (71.8% and 60.8%) and xylan (69.5% and 61.8%). It is clear from these results that the majority of the glucan initially present was recovered after IL pretreatment, and a significant portion of hemicellulose and lignin remained solubilized in the resultant IL-water mixture.

Compositional analysis of untreated forestry residue samples yielded 35.4% glucan, 10.1% mannan, 2.5% xylan, 3.5% galactan, 0.5% arabinan, 26.8% lignin and < 1% ash. Lower amounts of major polymeric sugars (glucan and mannan) coupled to the slightly increased amount of lignin suggests that the forestry residue samples were much more heterogeneous, likely containing plant materials other than Douglas-fir, bark, and a small percent of soil in their dry mass. Increasing pretreatment severity increased IL solubilization of hemicellulose and lignin while increasing the percentage of cellulose in the recovered biomass. At 120°C, 2 mm-milled forestry residues and un-milled forestry residues showed glucan recovery of 71.0% and 79.4%, respectively, while effectively removing major hemicellulose sugars such as mannan (57.5% and 61.1%, respectively), and xylan (21.1% and 41.4%, respectively). When pretreatment temperature was increased to 160°C the samples showed glucan recoveries of 62.8% and 82.4% with a concomitant reduction of hemicellulose observed in mannan (74.7% and 76.2%) and xylan (65.2% and 58.4%) for the 2 mm-milled forestry residues and un-milled residues, respectively. In all cases, total glucan recovery was higher for the un-milled samples, and this is most likely because of the higher mass recovery of these samples. For consistency, large intact twigs/woodchips were not included in compositional analysis or enzymatic saccharification experiments on un-milled samples. A control experiment was performed whereby un-milled Douglas-fir woodchips and forestry residues were subjected to acid hydrolysis and the glucan yields were less than 5%. Softwood wood contains approximately 66-72% polysaccharides while softwood bark contains only 30-48% polysaccharides [23], which may account for the lower yields of monomeric sugars observed during compositional analysis of the forestry residues.

Compositional analysis also revealed similar amounts of acid-insoluble lignin in untreated Douglas-fir woodchips (24.6%) and forestry residue samples (26.8%), and pretreatment with [C_2_mim][OAc] effectively removed lignin from both sets of samples. Wiley milling appeared to be a determining factor for lignin reduction in the Douglas-fir woodchips, while temperature was less important. For example, Douglas-fir woodchips pretreated at 120°C showed a 38.5% and a 13.5% reduction in lignin for 2 mm-milled and un-milled samples, respectively. At 160°C pretreatment conditions, the percent reduction of lignin of the 2 mm-milled and un-milled woodchips was 46.3% and 18.9%, respectively. Lignin removal from the forestry residue samples displayed a greater dependence on pretreatment severity, and was less influenced by Wiley milling. For example, 2 mm-milled samples at 120°C showed a 22.1% reduction in lignin while un-milled samples pretreated at the same temperature showed 18.4% reduction in lignin. Forestry residue samples pretreated at 160°C, however, showed a larger percent reduction of lignin, 29.0% and 25.8% for 2 mm-milled and un-milled samples, respectively. It has been shown that lignin removal efficiency is inversely proportional to biomass loading [7] and polarity of anti-solvent [3]. Although greater lignin removal can be accomplished at lower biomass loading [10,24] using organic solvent to precipitate cellulose, we selected 10% biomass loading and water as an antisolvent to minimize cost and environmental waste. Because softwood bark contains approximately 15% more lignin by mass [23], it is reasonable that the forestry residue samples lost approximately 7% more lignin than the Douglas-fir woodchips.

### Analysis of Douglas-fir samples after IL pretreatment and enzymatic saccharification

**Figure 2:**
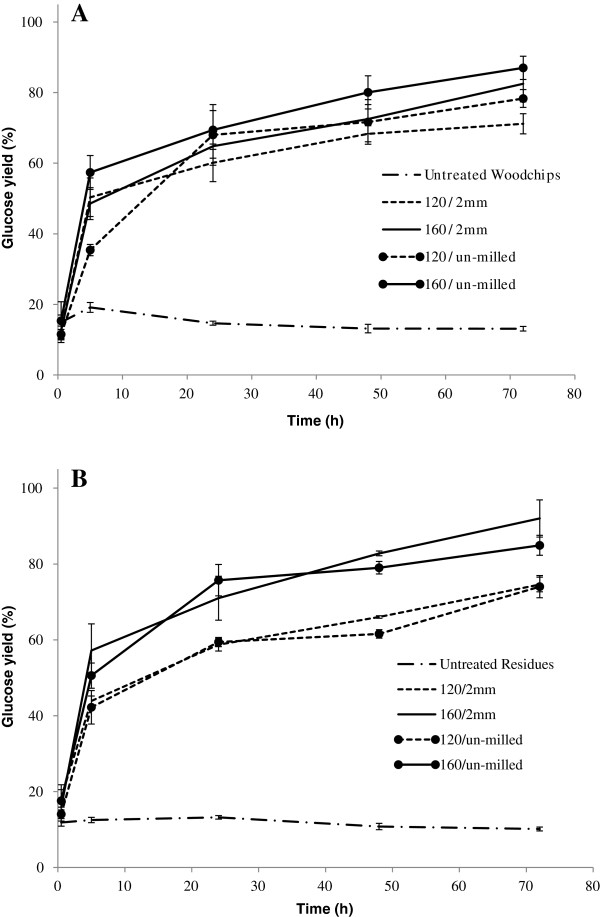
**Comparison of enzymatic saccharificaiton of untreated and ionic liquid pretreated Douglas-fir woodchips (A) and forestry residues (B).** Biomass loading = 100 g/L, enzyme loading = 20 mg CTec2 protein/g glucan and 4.25 mg HTec2 protein/g mannan.

Pretreatment with [C_2_mim][OAc] substantially increased enzymatic saccharification yields of both the Douglas-fir woodchips and forestry residues as compared to untreated controls. Biomass precipitated from IL pretreatment reactions with antisolvent was washed, lyophilized and used directly in enzymatic saccharification reactions. Figure 2 indicates that glucose yields from 160°C IL pretreated samples reached 82% and 87% for 2 mm-milled and un-milled samples of the Douglas-fir woodchips, respectively, after 72 hr of enzymatic saccharification. Samples pretreated at 120°C yielded slightly less glucose, 71% and 78%, from 2 mm-milled and un-milled samples, respectively. The forestry residue samples pretreated at 160°C also produced high glucose yields after enzymatic saccharification for 72 hr. The 2 mm-milled sample yielded 92% glucose as compared to the un-milled sample, which gave 85%. When samples were pretreated at 120°C saccharification yields were slightly lowered to 75% and 71% for 2 mm-milled and un-milled forestry residue samples, respectively. These data are not uncommon when comparing to other feedstocks, such as Eucalyptus, after IL pretreatment using [C_2_mim][OAc] [9,25].

### X-ray diffraction (XRD) of untreated and IL pretreated Douglas-fir samples

**Figure 3:**
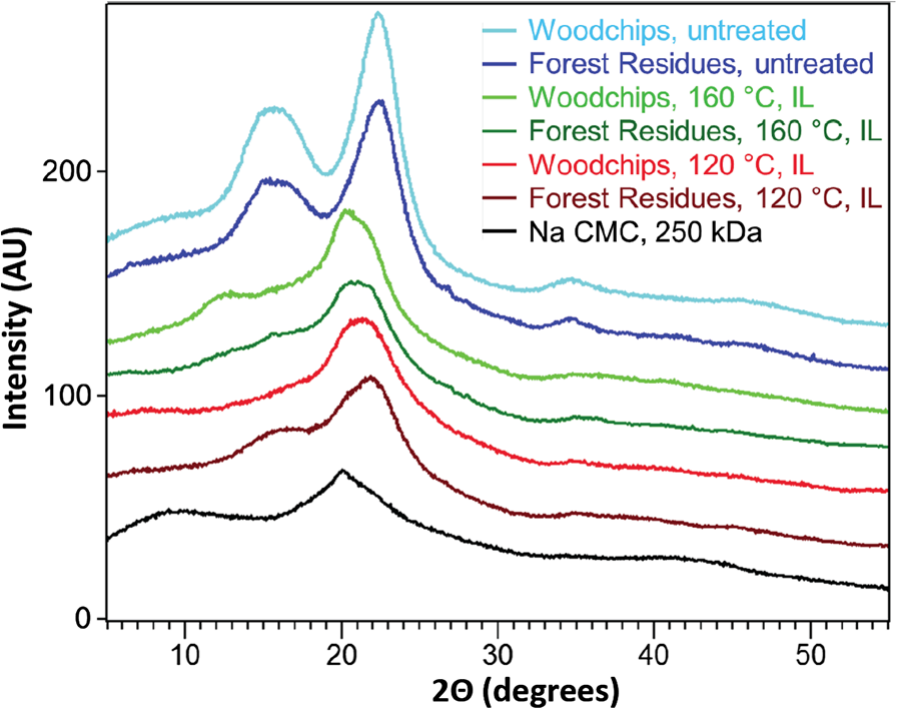
XRD patterns of samples used in this study and relative comparison with amorphous cellulose (Na CMC).

The XRD patterns of untreated and IL-pretreated woodchips and forestry residues as well as an amorphous control sample of sodium carboxymethyl cellulose (Na CMC) are shown in Figure 3. Because the biomass used in this study contained lignin and hemicellulose, crystallinity index (CrI) values can only be interpreted as relative comparisons. The untreated woodchips and forestry residue samples are crystalline with CrI values of 34% and 30%, respectively and show diffraction profiles characteristic of the cellulose I polymorph, with three major peaks at 35, 22 and 15-16° 2θ, corresponding to the [004], [200] and combined [110] + [1-10] lattice places, respectively. The most intense reflection (200) for the two untreated samples is observed at 22.3° (woodchips) and 22.1° 2θ (forestry residues). Upon IL pretreatment, the recovered biomass gave XRD patterns displaying significantly less ordered cellulose structures, as compared to the untreated samples. The major peaks at 22.3 and 22.1° 2θ shift to lower 2θ values (larger d-spacing). The combined peak at around 15-16° 2θ, as well as the peak corresponding to the [004] lattice plane in cellulose I at around 35° 2θ were reduced to undetectable levels. The only exception seems to be the forestry residue sample pretreated at 120°C, which displays a broad feature, centered around 16° 2θ. The major peaks in the XRD patterns of the woodchips and forest residue samples pretreated at 120°C are found around 21.0 and 21.8° 2θ; the corresponding peaks for the samples treated with IL at 160°C are shifted to 20.2 and 20.9° 2θ. The shifted position and distorted shape of the major reflection (200) in the IL-treated samples suggest that the cellulose structure is significantly distorted. The occurrence of a broad peak at about 12.5° 2θ in the XRD pattern of the woodchips sample pretreated at 160°C seems to indicate that small amounts of cellulose II may also be present. However, no such peak was detected in the XRD pattern of the 160°C IL treated forestry residue sample, suggesting the content of cellulose II in that material is extremely low or nonexistent [26].

The shift of the major reflection (200) position to lower 2θ values can be also explained by a distortion in the cellulose I structure. We recently proposed that such a distorted structure forms upon treating Avicel with [C_2_mim][OAc] and results in an expansion of the cellulose I lattice [27,28]. The CrI values of the untreated woodchips and forestry residue samples are 37% and 32%, respectively. The IL treated samples still display some residual crystallinity as evident from a comparison with a completely amorphous sample of Na CMC (Figure 3). Douglas-fir woodchips pre-treated at 120°C and 160°C displayed CrI values of 8 and 11%, respectively. Similarly, the forestry residues gave CrI values of 8% (120°C) and 10% (160°C). Pretreatment with IL in all cases lead to rather low CrI values indicating a loss of the native cellulose long-range crystallographic order. We hypothesize that the cellulose chains within the hydrogen-bonded sheets in the pretreated samples are essentially random, with some short-range order present in the direction perpendicular to the sheets that are attributed to the cellulose II polymorph or distorted cellulose I. Finally, we note that no significant differences between the XRD patterns of the IL pretreated 2mm-milled and unmilled samples are observed.

### Nuclear magnetic resonance studies of untreated and IL pretreated douglas-fir samples

**Figure 4:**
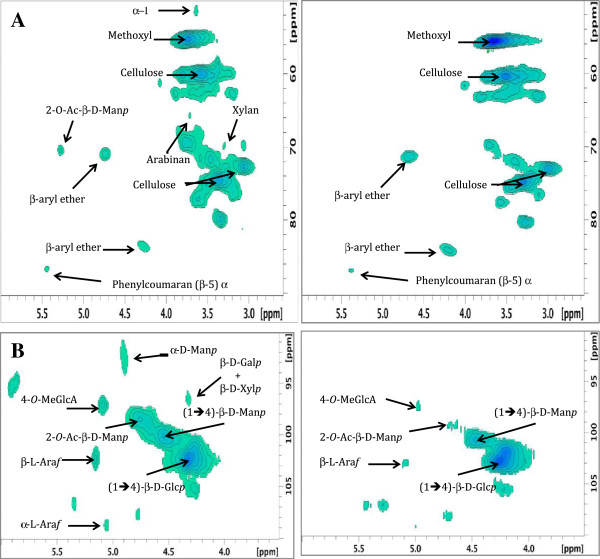
**(A) Lignin interunits, polysaccharide, and acylated polysaccharide regions of Untreated Douglas-fir Woodchips (left) and Pretreated Douglas-fir Woodchips (un-milled, 160°C, hr) (right).** (**B**) Polysaccharide anomeric region of Untreated Douglas-fir Woodchips (left) and Pretreated Douglas-fir Woodchips (un-milled, 160°C, hr) (right).

**Figure 5:**
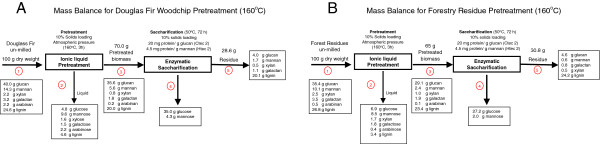
**(A) Mass balance for Douglas-fir woodchip and (B) un-milled forestry residue pretreatments at 160°C.**

Nuclear Magnetic Resonance (NMR) studies of untreated Douglas-fir woodchips revealed lignin interunit, polysaccharide, acylated polysaccharide and anomeric resonances similar to pine [29]. Both untreated and IL pretreated Douglas-fir woodchips showed large cross peaks that can readily be assigned to lignin methoxyl groups *δ* (^1^H/^13^C) 3.73/55.4, and cellulose (*δ* 3.49-3.79/60.2, *δ* 3.37/74.3, *δ* 3.07/72.6) indicating that these polymers were not extensively removed during pretreatment (Figure 4). β-aryl ether crosspeaks at *δ* 4.73/71.0 and *δ* 4.27/83.8 as well as the phenylcoumaran (β-5) α resonance (*δ* 5.44/86.7) also appeared in both spectra indicating that these linkages remain intact during pretreatment. A series of correlations corresponding to minor components of Douglas-fir hemicellulose, such as those for xylan (*δ* 3.30/70.1), β-D- xylopyranosyl (*δ* 4.32/96.8), arabinan (*δ* 3.71/65.8), and α-L-arabinofuranosyl (*δ* 5.05/108.8) were absent from the spectrum of the pretreated material. Several additional hemicellulose resonances were assigned to mannan, the major hemicellulose component of Douglas-fir. For example, a 2-O-acyl-β-D-mannopyanosyl correlation (*δ* 5.38/70.6) was also absent from the pretreated biomass spectrum, while a second resonance assigned to 2-O-acyl-β-D-mannopyanosyl polymer (*δ* 4.77/98.6) was significantly decreased in the pretreated spectrum (Figure 5). Another large cross peak in the untreated spectrum that was missing from the pretreated material corresponded to the α-D-mannopyrosyl resonances at *δ* 4.88/92.5. Interestingly, the α -1 atom (*δ* 3.64/51.4), identified in Douglas-fir lignin by Berlin et al. [30], is clearly missing after pretreatment, suggesting scission of the bond in the α – 1 linkage occurs during pretreatment with [C_2_mim][OAc] at the conditions studied.

### Mass balance of IL pretreatment

A detailed mass balance for un-milled Douglas-fir woodchips and forestry residues pretreated 160°C is presented in Figure 5. At 160°C, based on 100 g of dry weight, 70 g of solids were recovered from un-milled Douglas-fir woodchips and retained 91% of the glucan. Similar results were obtained for the forestry residues at this temperature, whereby 65 g of solids were recovered after pretreatment, representing 82% of the original glucan. As compared to other IL pretreatment studies, a significant fraction of the lignin was retained for the woodchips and forestry residues after IL pretreatment (81% and 87%, respectively), presumably due to the higher biomass loading used in this report. Following enzymatic saccharification of the un-milled samples pretreated at 160°C, approximately 30% of the material remained undigested. Of this material approximately 75% was identified as lignin by compositional analysis using the NREL protocol [31]. After pretreatment at 120°C, greater amounts of residue were obtained after enzymatic hydrolysis for both Douglas-fir (49.1% increase) and forestry residues (53.2% increase). This higher mass recovery is partially due to increased amounts of recalcitrant cellulose as evidenced by the higher amounts of glucan in the samples. The overall glucan closure for Douglas-fir woodchips was 93.5% while that of the un-milled forestry residues was 91.0% suggesting that less than 10% of glucose was lost during pretreatment of samples at 160°C. The overall glucan closures for Douglas-fir woodchips and un-milled forestry residues pretreated at 120°C were 97.4% and 96.1%, respectively. Both Douglas-fir woodchip and forestry residue samples pretreated at 120°C showed slightly higher masses in the post-enzymatic saccharification solid residue, which can be accounted for by undigested glucan and hemicellulose. A detailed mass balance of un-milled Douglas-fir woodchips and forestry residues pretreated 120°C is presented in the Additional file 1: Figures S1 and Additional file 2: Figure S2 (Figure 2).

## Conclusions

The majority of forestland in the United States is owned by private forestry industry, and approximately 368 million dry tons of forestry residue biomass is available for harvesting in the Unites States annually. Though only 142 million dry tons of this material is actually harvested, these residues account for approximately 50% of current biomass energy consumption in the United States and will continue to be an important feedstock for future production of biofuels [32].

Although pretreatment of softwoods with imidazolium-based ILs has been investigated previously, this is the first study demonstrating the utility of ionic liquid pretreatment and subsequent enzymatic saccharification of Douglas-fir wood chips and forestry residues. With glucose yields between 70-90% for three-hour pretreatments at 120°C and 160°C, our results compare well to those obtained from pine [5]. As compared to dilute sulfuric acid or SO_2_ steam explosion of Douglas-fir, our methods produce equivalent glucose yields. Ionic liquid pretreatment of softwood forest residuals, therefore, adds to a growing base of knowledge for utilizing forestry and agricultural residues for biofuel production [12].

The data shows that 160°C pretreatment of Douglas-fir woodchips provides only 5% increase in glucose yields from enzymatic saccharification as compared to pretreatment at 120°C. The energy savings, and near two-fold increase in mannose yields, obtained by the milder severity outweighs this slight debt of glucose. Forestry residues were found to contain approximately 5% less glucan and 4% less mannan, than the Douglas-fir woodchips, but at $47/dry ton of biomass the residues represent a more attractive feedstock than the woodchips ($125/ dry ton of biomass) [33].

Our results also indicate no statistically significant increase in sugar yields from Wiley-milling the Douglas-fir woodchips occurs at 120°C or 160°C. We attribute these results to physical penetration of the ionic liquid into the plant cell walls allowing the disruption of the hydrogen bond matrix of crystalline cellulose [34]. A study performed on switchgrass and hardwood feedstocks showed that hammer-milling of the former required approximately 52 kWh/tonne while knife-milling of the latter required 50 kWh/tonne to obtain particle sizes of 2 mm and 4 mm, respectively [35].

Combined, the data suggest that future biorefineries can cut costs by processing forestry residuals with minimal mechanical milling.

## Methods

### Feedstock and ionic liquid pretreatment

The feedstocks used in this study consisted of Douglas-fir (*Pseudotsuga menziesii*) wood chips from primary forest products manufacturing (sawmill residue chips etc.) accumulated from across the southwest Washington region, and forestry residues harvested from industrial private Douglas-fir timberlands in northwest Oregon. The Douglas-fir wood chips were chipped to approximately 1-4 cm × 1-4 cm × 0.3-0.6 cm using a variety of mill residue disk chippers, producing a typical “pulp mill furnish” chip. The forest residues were more heterogeneous, ranging in size from approximately 2-10 cm × 1-5 cm × 0.2–1 cm, as they were produced from a mobile horizontal grinder / chipper at a timber harvest landing in the woods. Both sets of chip samples were screened over a gyratory screen with a woven-wire mesh bottom screen with 3 mm openings to remove the fine particles. The two sets of retained “accept” samples were pretreated without any additional processing, and are referred to hereafter as “un-milled” (Figure 1). A subset of the same samples (i.e. Douglas-fir woodchips and forestry residues) were ground to a 2 mm mesh size using a Thomas Model 4 Wiley® Mill machine. These samples are referred to as “2 mm-milled” in the subsequent text (Figure 1).

The samples were pretreated using the IL, 1-ethyl-3-methylimidazolium acetate, abbreviated hereafter as [C_2_mim][OAc], purchased from BASF, and used without additional purification. Samples of Douglas-fir Woodchips and Forestry Residues were individually pretreated with [C_2_mim][OAc] at either 120°C or 160°C for 3 h, under nitrogen, in an automated 1L Globe oil jacketed reactor system (Syrris, Inc., Charlestown, MA). Ten percent biomass loading was achieved by using 30 g of dry biomass in 270 g of [C_2_mim][OAc] which was allowed to stand overnight (25°C, 18 h) prior to heating. Though an IL pre-incubation allowed for slightly better mixing, samples that were not pre-incubated overnight did not show significant differences in sugar yields after enzymatic saccharification. Pretreatment reactions were conducted in triplicate with constant stirring at 315 RPM using 80 mm diameter PTFE anchor-type impeller, powered by a Heidolph RZR 2052 mechanical stirrer (Heidolph Instruments GmbH & Co. KG, Schwabach, Germany). Pretreatment reactions were quenched with 900 mL of tap water as an antisolvent to precipitate biomass used for compositional analysis and enzymatic saccharification. The resulting IL/water/biomass mixture often formed a gel and was therefore blended (Waring® Commercial Laboratory Blender, 3 × 3 second pulses) before filtering through a fine stainless steel mesh. The recovered biomass was washed four additional times, each with 900 mL of tap water, to remove any residual IL. The recovered solids were lyophilized in a FreeZone Freeze Dry System (Labconco, Kansas City, MO) and used for compositional analysis, enzymatic saccharification, XRD and NMR studies.

### Compositional analysis of untreated and IL pretreated Douglas-fir

Total sugars and acid-insoluble lignin from Douglas-fir woodchips and forestry residues were determined according to the two-step acid hydrolysis procedure from the National Renewable Energy Laboratory [31]. Briefly, 300 mg of sample and 3 mL of 72% H_2_SO_4_ was added to a 100 mL serum bottle and incubated at 30°C with stirring at 175 RPM for 1 hr. The sample was then diluted to 84 mL with deionized water and autoclaved for 1 hr. Following hydrolysis, 1 mL of each sample was neutralized with 80 mg of CaCO_3_ and spin-filtered through 0.45 micron Whatman Unifilter® 96-well plate PVDF filters. Monomeric sugars were analyzed on an Agilent 1200 HPLC using an isocratic aqueous mobile phase of 0.6 mL/min, while maintaining a 7.8 × 300 mm Aminex® HPX-87P (Bio-Rad) analytical column at 85°C [17]. All acid hydrolysis reactions were run in triplicate, and quantified using a 3-point calibration curve with R^2^ value of 0.99. The results from compositional analysis of all pretreated samples and untreated controls are summarized in Table 1.

### X-ray diffraction measurements

XRD data were collected with a PANalytical Empyrean X-ray diffractometer equipped with a PIXcel^3D^ detector and operated at 45 kV and 40 kA using Cu *Kα* radiation (λ = 1.5418 Å). The patterns were collected in the 2θ range of 5 to 55°, the step size was 0.026°, with an exposure time of 600 seconds. A reflection-transmission spinner was used as a sample holder and the spinning rate was set at 8 rpm throughout the experiment. The crystallinity index (CrI) was determined from the ratio of the crystalline peak area to the total area using the software package HighScore Plus ®.

### Enzymatic saccharification of IL pretreated Douglas-fir

Enzymatic saccharification of pretreated biomass samples and untreated biomass controls were run in triplicate according to the NREL Laboratory Analytical Procedure “Enzymatic Saccharification of Lignocellulosic Biomass”. Solid and enzyme loading concentrations were based upon previously optimized experiments for dilute acid pretreatment [17]. All reactions were run in an Enviro-Genie® (Scientific Industries, Inc.) rotisserie incubator at 50°C with 10% solids loading by suspending 1.0 g of lyophilized biomass in 10.0 mL of 0.05 M citrate buffer (pH 4.8) containing 0.1% w/v solution of sodium azide. Cellulase enzyme loading of 20 mg CTec2 (Batch# VCN10001, protein content 188 mg/mL) per gram of glucan and hemicellulase enzyme loading of 5 mg HTec2 (Batch# VHN00001, protein content 27 mg/mL) per gram of mannan were calculated from compositional analysis data. The enzyme cocktails were gifts from Novozymes N.A. (Franklin, NC). Samples were collected at 0.5, 5, 24, 48 and 72 hr timepoints, spin-filtered, and diluted 10-fold with deionized water. Samples were analysed using identical conditions as those described for compositional analysis. Glucose yield was defined as the amount of glucose obtained by enzymatic saccharification divided by the total maximum glucose amount available in pretreated biomass samples or untreated biomass controls as obtained by compositional analysis. After enzymatic saccharification, the remaining solids were washed with 3 × 30 mL of deionized water, centrifuged after each wash to remove any residual monomeric sugars, and then lyophilized. The dry material was analyzed for glucan, mannan and acid-insoluble lignin using the NREL protocol used for compositional analysis [31]. The results completed the mass balance presented in Figure 4 (Additional file 1: Figure S1 and Additional file 2: Figure S2).

### Nuclear magnetic resonance

Two-dimensional nuclear magnetic resonance (NMR) spectroscopy was performed according to protocols developed for whole plant cell wall characterization [29,36] on a Bruker instrument (600 MHz for ^1^H) equipped with an inverse cryoprobe. Untreated, 2 mm-milled, Douglas-fir woodchips and un-milled, 160°C pretreated Douglas-fir woodchips were lyophilized, and 200 mg of each sample was ball milled (Retsch, PM-100) for 5 × 20 min intervals, with 10 min pauses between milling to avoid overheating. Samples (50 mg) were carefully poured into a 5 mm NMR tube, to which was added 750 μL of DMSO-*d*_*6*_ and the resulting mixture was sonicated for 3-5 hr to produce a homogeneous gel. The HSQC adiabatic-pulse program hsqcetgpsisp.2 was used with f2 (proton) acquisition time of 142 ms and f1 (carbon) acquisition time of 3.8 ms. D1 was set to 1.5 sec, TD was set to 2048 (f2) and 256 (f1). Data processing was accomplished using Bruker Topspin 3.1 software (Macintosh) using QSINE apodization, GB = 0.001, and line broadening for of –0.05Hz (f2) and -0.1 Hz (f1). Signal contour intensities were manually matched for spectral comparison in Figures 4 and 5. Cross peaks were referenced to residual solvent signal for DMSO at *δ*^1^H/^13^C = 2.50/39.5 ppm.

## Abbreviations

[C2mim][OAc]: 1-ethyl-3-methylimidazolium acetate; NMR: Nuclear magnetic resonance; XRD: X-Ray Diffraction; HPLC: High-performance liquid chromatography.

## Competing interests

The authors declare that they have no competing interests.

## Authors’ contributions

SS and BAS (Supervisors) conceived of this study and edited the manuscript. AMS conducted pretreatment, compositional analysis, enzymatic saccharification, HPLC and NMR experiments and wrote the manuscript. SP assisted with compositional analysis and HPLC sample preparation. VS performed all XRD studies and contributed to the XRD sections of the manuscript. All authors read and approved the final manuscript.

## Supplementary Material

Additional file 1: Figure S1 Structure of lignin α – 1 linkage. Click here for file

Additional file 2: Figure S2 Mass balances for Douglas-fir woodchip and un-milled Forestry Residue Pretreatments at 120°C. Click here for file
