# Supplementary material for: Comparison of sugar content for ionic liquid pretreated Douglas-fir woodchips and forestry residues
Source: Biotechnol Biofuels. 2013 May 1;6:61. doi: 10.1186/1754-6834-6-61 (PMC3672072; doi:10.1186/1754-6834-6-61)
Supplement: Additional file 1: Figure S1 — Structure of lignin α – 1 linkage. [file 1754-6834-6-61-S1.docx]

**Supplementary Materials:**

**Figure 1:** Structure of lignin α linkage
