# Supplementary material for: Comparison of sugar content for ionic liquid pretreated Douglas-fir woodchips and forestry residues
Source: Biotechnol Biofuels. 2013 May 1;6:61. doi: 10.1186/1754-6834-6-61 (PMC3672072; doi:10.1186/1754-6834-6-61)
Supplement: Additional file 2: Figure S2 — Mass balances for Douglas-fir woodchip and un-milled Forestry Residue Pretreatments at 120°C. [file 1754-6834-6-61-S2.docx]

**Supplementary Materials:**


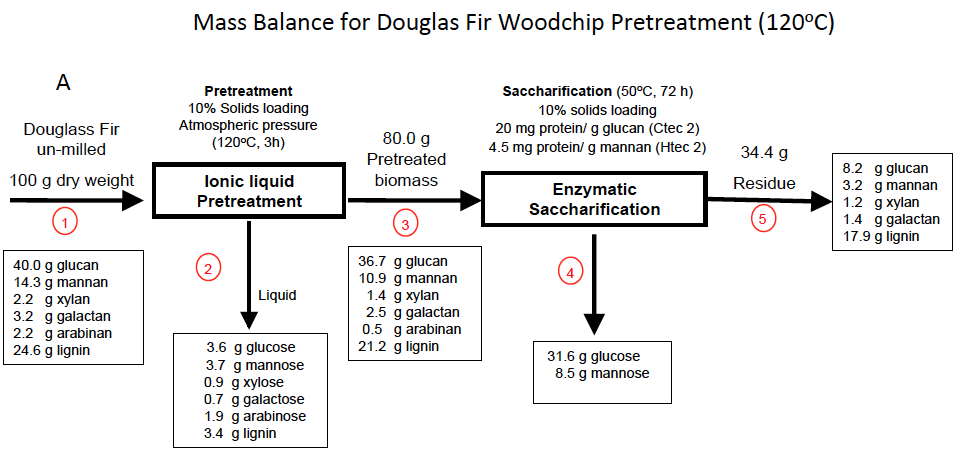


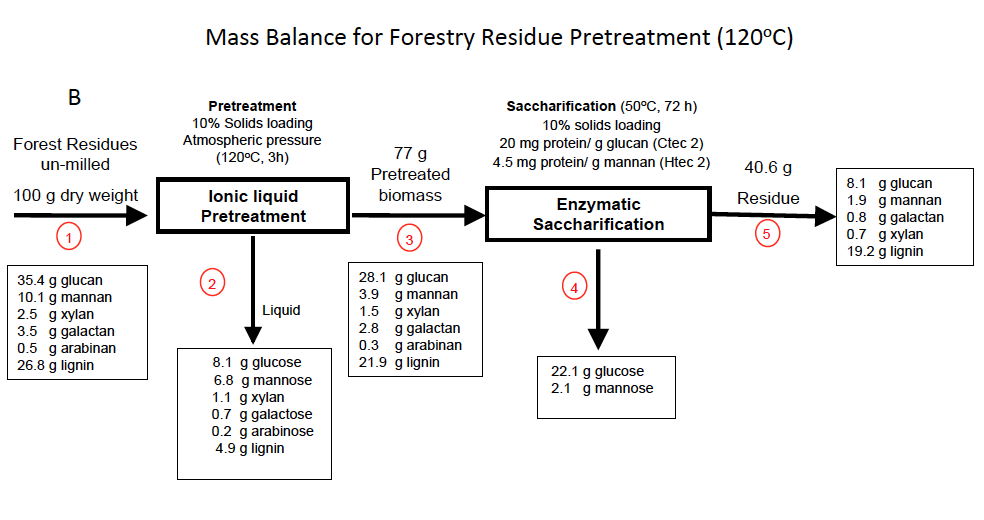


**Figure 2:** Mass balances for Douglas-fir woodchip and un-milled Forestry Residue Pretreatments at 120 ^o^C.
